# Supplementary material for: Beyond Infant Nutrition: Investigating the Long-Term Neurodevelopmental Impact of Breastfeeding
Source: Nutrients. 2025 Aug 8;17(16):2578. doi: 10.3390/nu17162578 (PMC12389622; doi:10.3390/nu17162578)
Supplement: Supplementary file 1 [file nutrients-17-02578-s001.zip › nutrients-3706317-supplementary.pdf]

**Supplementary material 1:** Partial Correlation Results at 5 years.

| | | Motor Development | Language development | Articulation | Nonverbal intelligence | Behavior | Total developmental score |
|---|---|---|---|---|---|---|---|
| **Breastfeeding duration** | Spearman's rho | 0.012 | -0.050 | 0.034 | 0.118 | -0.108 | 0.008 |
| | p-value | 0.911 | 0.649 | 0.759 | 0.283 | 0.327 | 0.945 |
